# The Inhibitory Effect of Cordycepin on the Proliferation of MCF-7 Breast Cancer Cells, and Its Mechanism: An Investigation Using Network Pharmacology-Based Analysis

**DOI:** 10.3390/biom9090414

**Published:** 2019-08-26

**Authors:** Dahae Lee, Won-Yung Lee, Kiwon Jung, Yong Sam Kwon, Daeyoung Kim, Gwi Seo Hwang, Chang-Eop Kim, Sullim Lee, Ki Sung Kang

**Affiliations:** 1 School of Pharmacy, Sungkyunkwan University, Suwon 16419, Korea; 2 College of Korean Medicine, Gachon University, Seongnam 13120, Korea; 3 Institute of Pharmaceutical Sciences, College of Pharmacy, CHA University, Sungnam 13844, Korea; 4 Dong-A Pharmaceutical Co., LTD., Yongin 17073, Korea; 5 Department of Life Science, College of Bio-Nano Technology, Gachon University, Seongnam 13120, Korea

**Keywords:** *Cordyceps militaris*, brown rice, cordycepin, MCF-7, apoptosis

## Abstract

*Cordyceps militaris* is a well-known medicinal mushroom. It is non-toxic and has clinical health benefits including cancer inhibition. However, the anticancer effects of *C. militaris* cultured in brown rice on breast cancer have not yet been reported. In this study, we simultaneously investigated the anticancer effects of cordycepin and an extract of *C. militaris* cultured in brown rice on MCF-7 human breast cancer cells using a cell viability assay, cell staining with Hoechst 33342, and an image-based cytometric assay. The *C. militaris* concentrate exhibited significant MCF-7 cell inhibitory effects, and its IC_50_ value was 73.48 µg/mL. Cordycepin also exhibited significant MCF-7 cell inhibitory effects, and its IC_50_ value was 9.58 µM. We applied network pharmacological analysis to predict potential targets and pathways of cordycepin. The gene set enrichment analysis showed that the targets of cordycepin are mainly associated with the hedgehog signaling, apoptosis, p53 signaling, and estrogen signaling pathways. We further verified the predicted targets related to the apoptosis pathway using western blot analysis. The *C. militaris* concentrate and cordycepin exhibited the ability to induce apoptotic cell death by increasing the cleavage of caspase-7 -8, and -9, increasing the Bcl-2-associated X protein/ B-cell lymphoma 2 (Bax/Bcl-2) protein expression ratio, and decreasing the protein expression of X-linked inhibitor of apoptosis protein (XIAP) in MCF-7 cells. Consequently, the *C. militaris* concentrate and cordycepin exhibited significant anticancer effects through their ability to induce apoptosis in breast cancer cells.

## 1. Introduction

The death rate due to breast cancer is 14%, and this malignancy accounts for 23% of all cancer cases. Therefore, it remains a significant clinical and societal issue worldwide [1]. Patients diagnosed with breast cancer may receive drug therapy, surgery, hormone therapy, and/or radiotherapy. These treatments have a significant and long-term impact on patients’ lives owing to the side effects of drug therapy and hormone therapy and the limitations of surgery and radiotherapy [2,3]. Among them, cisplatin is one of the most widely used chemotherapeutic drugs for clinical treatment [4]. Although the use of cisplatin causes toxic adverse effects such as nephrotoxicity, this drug is still widely used due to its high efficacy as an anticancer drug [5]. For this reason, interest in the development of drugs from natural products for the prevention and treatment of breast cancer has increased. Experimental studies indicate that many natural products and their bioactive compounds have anticancer effects on breast cancer [6,7,8,9,10]. Various mechanisms, including inhibition of cell proliferation, [6,11] apoptosis [11], cell cycle arrest, inhibition of metastasis [6], downregulation of estrogen receptor alpha (ER-α) expression [7], and inhibition of angiogenesis of cells, are involved in anticancer effects on breast cancer [3]. Therefore, researchers in the past have urged continuing research on potential and effective natural products so as to identify their bioactive compounds for the treatment of breast cancer. Medicinal mushrooms such as *Cordyceps* species, *Agaricus bisporus*, *Pleurotus eous*, *Ganoderma lucidum*, and *Amauroderma rude* have shown anticancer effects on breast cancer both in vivo and in vitro [7,12]. Among medicinal mushrooms, *Cordyceps* species are considered to have various positive aspects in terms of safety (nontoxicity) [13] as well as clinical health effects such as immuno-enhancing activity [11], neuroprotective activity [14,15], anticancer effects [16], antimicrobial activity [17], and anti-inflammatory activity [18,19]. Many experimental studies have also been published on the anticancer effects of extracts of *C. militaris* in vivo and in vitro on breast cancer [20,21,22,23].

However, *Cordyceps* species are very rare in nature and are difficult to commercialize. Therefore, studies on *Cordyceps* species are not consistent because they have been studied using mycelia, wild-collected specimens, and *Cordyceps* cultured in insects, and other sources. Recently, the process of cultivating *Cordyceps* species in brown rice was successfully established, and many studies using *Cordyceps* species cultured in brown rice have been reported [18,24,25,26,27,28,29]. Among these, *Cordyceps militaris*, which is known as a representative *Cordyceps* species, has been cultured in brown rice, and its extract has been reported to have anticancer effects, including effects against breast cancer [25].

Many compounds such as cordycepin, mannitol, ergosterol, and polysaccharides isolated from *C. militaris* have been reported to have diverse pharmacological activities with anti-oxidative, anti-inflammatory, antiviral, antidiabetic, anti-platelet aggregation, and anticancer effects [30,31,32]. In particular, the anticancer effects of cordycepin on various cancers have been reported in many studies [20,33,34]. These anticancer effects of cordycepin are attributed to a structural similarity with cellular nucleosides and adenosine. Thus, cordycepin acts like a nucleoside analogue and inhibits the polyadenylation of mRNA of cancer cells [35,36]. While cordycepin has been examined in a number of studies related to cancer, there are no studies simultaneously examining the anticancer effects of cordycepin and *C. militaris* on breast cancer.

Network pharmacology has emerged as a promising approach to elucidate the systems-level mechanisms of natural products [37,38,39]. It understands complex diseases, including cancer, as a perturbation of interconnected complex biological networks and identifies the mechanism of drug action in terms of the network topology [40,41]. Natural products are known to exert their therapeutic effects by acting on multiple targets of multiple ingredients, and these mechanisms are well-suited to the core concept of network pharmacology. Therefore, researchers have employed network pharmacology methods to screen potent anticancer agents from natural products by predicting their potential targets and pathways [41,42,43].

In the present study, we investigated the anticancer effects of cordycepin and the extract of *C. militaris* cultured in brown rice on MCF-7 human breast cancer cells. To explore the systems-level mechanism of cordycepin, we predicted potential targets and pathways related to breast cancer using network pharmacology methods. Finally, we verified the predicted targets of cordycepin related to the apoptosis pathway in vitro.

## 2. Materials and Methods

### 2.1. Cordyceps Militaris Concentrate

*Cordyceps militaris* concentrate was provided by Dong-A Pharmaceutical (Yongin, Korea). Briefly, it was extracted in 50% ethanol (*v*/*v*) solution in water from *Cordyceps militaris* cultured in brown rice. Then, it was concentrated under low pressure. For the in vitro and in vivo experiments, it was freeze-dried.

### 2.2. Cell Culture

The MCF-7 human breast cancer cell line, MDA-MB-231 human breast cancer cell line, LLC-PK1 pig kidney epithelial cell line and human umbilical vein endothelial (HUVEC) cell line were purchased from the American Type Culture Collection (ATCC, Manassas, VA, USA). MCF-7 and MDA-MB-231 cells were grown in Roswell Park Memorial Institute 1640 medium (RPMI 1640) (Corning, Manassas, VA, USA). LLC-PK1 cells were grown in Dulbecco’s modified Eagle’s medium (Corning, Manassas, VA, USA). Both media types contained 10% fetal bovine serum (Gibco BRL, Carlsbad, MD, USA), 100 units/mL penicillin, and 100 mg/mL streptomycin. HUVECs were grown in Clonetics EGM-2 MV Microvascular Endothelial BulletKit (Lonza Inc., Walkersville, MD, USA). Cultures were maintained at 37 °C in a humidified atmosphere with 5% CO_2_.

### 2.3. Determination of Cell Viability

The cell viability of MCF-7 cells in *C. militaris* concentrate and cordycepin was evaluated using an Ez-Cytox Cell Viability Assay Kit (Dail Lab Service Co., Seoul, Korea). Briefly, cells with a density of 1 × 10^4^ cells/100 µL were seeded onto 96-well plates. After incubation for 24 h, *C. militaris* concentrate and cordycepin at various concentrations were added. After treatment for 24 h, 10 µL of Ez-Cytox solution was added and incubated for 30 min. The absorbance was measured at 450 nm (absorbance for live cells) in a microplate reader (PowerWave XS; Bio-Tek Instruments, Winooski, VT, USA). Cisplatin, which is one of the most widely used chemotherapeutic drug for breast cancer, was used as a positive control.

### 2.4. Cell Staining with Hoechst 33342

The nuclear condensation of MCF-7 cells induced by *C. militaris* concentrate and cordycepin was evaluated using Hoechst 33342 staining (Sigma Aldrich, St. Louis, MO, USA). Briefly, cells at a density of 4 × 10^5^ cells/3 mL were seeded onto 6-well plates. After incubation for 24 h, *C. militaris* concentrate and cordycepin at various concentrations were added. After treatment for 24 h, 2 μL of Hoechst 33342 solution was added, and the plates were then incubated for 10 min. Subsequently, the stained cells were analyzed using an IX50 fluorescent microscope equipped with a charge-coupled device (CCD) camera (Olympus, Tokyo, Japan).

### 2.5. Image-Based Cytometric Assay

The apoptotic death of MCF-7 cells induced by *C. militaris* concentrate and cordycepin was evaluated using an image-based cytometric assay kit (Invitrogen, Temecula, CA, USA). Briefly, cells at a density of 4 × 10^5^ cells/3 mL were seeded onto 6-well plates. After incubation for 24 h, *C. militaris* concentrate and cordycepin at various concentrations were added. After treatment for 24 h, cells were collected and suspended in Annexin V Binding Buffer (Life Technologies, Carlsbad, CA, USA). Then, Annexin V Alexa Fluor 488 (Invitrogen) was added and the cells were incubated in the dark for 30 min at 20 ± 5 °C. Next, apoptotic cells stained with green Annexin V Alexa Fluor 488 were counted and analyzed with a Tali image-based cytometer (Invitrogen).

### 2.6. Western Blot Analysis

The signaling pathways of MCF-7 cell apoptosis induced by *C. militaris* concentrate and cordycepin were evaluated using western blot analysis. Briefly, cells at a density of 4 × 10^5^ cells/3 mL were seeded onto 6-well plates. After incubation for 24 h, *C. militaris* concentrate and cordycepin at various concentrations were added. After treatment for 24 h, cells were collected using a cell scraper and lysed using radio immunoprecipitation assay (RIPA) buffer (Cell Signaling Technology, Inc., MA, USA) containing 1× ethylenediaminetetraacetic acid (EDTA)-free protease inhibitor cocktail and 1 mM phenylmethylsulfonyl fluoride (PMSF). The protein concentrations of samples were determined using a Pierce BCA Protein Assay Kit (Thermo Scientific, Carlsbad, CA, USA). Protein (20 μP) was mixed with 4× NuPAGE LDS Sample Buffer (Thermo Scientific, Carlsbad, CA, USA). After boiling for 10 min, the proteins were separated on precast 4–15% Mini-PROTEAN TGX (Tris-Glycine. eXtended) gel (Bio-Rad, Hercules, CA, USA) and then electrotransferred onto polyvinylidene fluoride (PVDF) transfer membranes. To enhance the detection of proteins, membranes were incubated with specific primary antibodies to Bcl-2-associated X protein (Bax), B-cell lymphoma 2 (Bcl-2), cleaved caspase-7, cleaved caspase-8, X-linked inhibitor of apoptosis protein (XIAP), and glyceraldehyde 3-phosphate dehydrogenase (GAPDH) (Cell Signaling Technology, Inc.), followed by incubation with horseradish peroxidase-conjugated secondary goat anti-rabbit antibody (Cell Signaling Technology, Inc.). The bound antibodies were visualized using Pierce ECL (enhanced chemiluminescence) Western Blotting Substrate (Rockford, IL, USA) and a FUSION Solo Chemiluminescence System (PEQLAB Biotechnologie GmbH, Erlangen, Germany).

### 2.7. Network Pharmacological Analysis

Network pharmacological analysis was performed by predicting targets of ingredients in traditional Chinese medicine (TCM) and constructing a compound–target network. The potential targets of cordycepin were obtained from the TCM-mesh (http://mesh.tcm.microbioinformatics.org) based on the combined score [44]. The combined score is calculated by a model that predicts drug–target interaction using random forests [45]. To get as many potential targets of cordycepin as possible, we set the threshold of the combined score to 150 (minimal threshold suggested by TCM-mesh). Then, a compound–target network was constructed by linking herbal ingredients and their predicted targets using the drug–target interaction information.

Gene set enrichment analysis (GSEA) based on the Kyoto Encyclopedia of Genes and Genomes (KEGG) database was performed to identify potential pathways related to breast cancer using Enrichr [46]. Enrichr computes enrichment by assessing multiple gene-set libraries (e.g., gene ontology, KEGG, and Online Mendelian Inheritance in Man (OMIM)) and calculates adjusted *p*-values, Z-scores and combined scores for the gene lists of interest (target genes). The combined score is calculated by the logarithm of multiplication of the *p*-value and z-score (note that this combined score is different from the combined score in the TCM-mesh).

### 2.8. Statistical Analysis

The data are presented as the mean ± standard deviation (SD). Statistical significance was determined using the Student’s *t*-test and the hypergeometric test. *p*-Values less than 0.05 were considered statistically significant.

## 3. Results

### 3.1. Effects of Cordyceps Militaris Concentrate and Cordycepin on MCF-7 Breast Cancer Cell Viability

**Figure 1:**
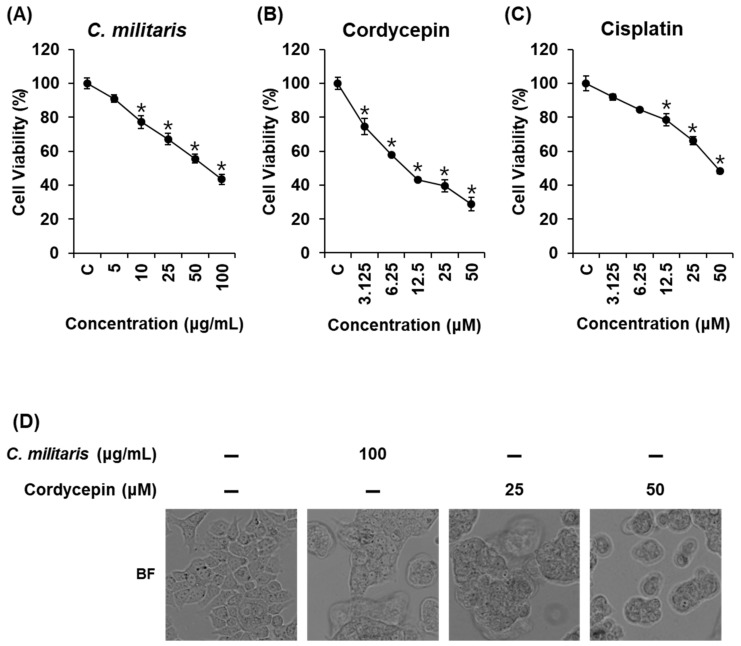
Effects of *Cordyceps militaris* concentrate and cordycepin on MCF-7 breast cancer cell viability. Cytotoxic effects of (**A**) *C. militaris* concentrate, (**B**) cordycepin, and (**C**) cisplatin on MCF-7 cells. (**D**) Effects of *C. militaris* concentrate and cordycepin on morphological changes in MCF-7 cells. Data are the means of experiments performed in triplicate. Data are presented as the mean ± standard deviation (SD). and were analyzed using the Student’s *t*-test. *p < 0.05 versus non-treated cells.

Cell viability assays were performed to evaluate the cytotoxic effects of the *C. militaris* concentrate and cordycepin on MCF-7 cells. Cisplatin was used as the positive control. *C. militaris* concentrate suppressed cell proliferation in a concentration-dependent manner (Figure 1A, IC_50_: 73.48 ± 2.76 µg/mL). Cordycepin also significantly suppressed cell proliferation in a concentration-dependent manner (Figure 1B, IC_50_: 9.58 ± 0.99 µM). Cisplatin suppressed cell proliferation in a concentration-dependent manner, but its IC_50_ value was higher than that of cordycepin (Figure 1C, IC_50_: 48.50 ± 1.84 µM).

Based on these results, we investigated the effects of *C. militaris* concentrate and cordycepin on morphological changes, which contributed to the inhibition of MCF-7 cell viability, using a phase contrast inverted microscope and phase contrast fluorescence microscope (Figure 1D). After treatment with *C. militaris* (100 µg/mL) and cordycepin (25, 50 µM), most cells detached from the cell culture plate, and apoptotic cell morphology such as membrane blebbing, cell shrinkage, and cell condensation [47] increased in comparison to the untreated normal cells.

### 3.2. Network Pharmacological Analysis of Cordycepin

**Figure 2:**
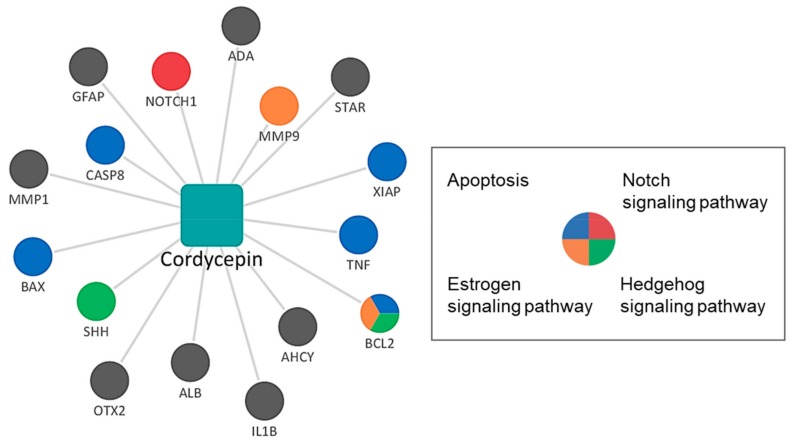
Compound–target network of *Cordyceps militaris*. Rectangles represent the compounds, and circles represent the targets. Nodes related to apoptosis, estrogen, hedgehog, and notch signaling pathway in KEGG and validated targets by western blotting analysis are colored. ADA, adenosine deaminase; AHCY, adenosylhomocysteinase; ALB, albumin; BAX, BCL2 associated X, apoptosis regulator; BCL2, BCL2 apoptosis regulator; CASP8, caspase 8; GFAP, glial fibrillary acidic protein; IL1B, interleukin 1 beta; MMP1, matrix metallopeptidase 1; MMP9, matrix metalloproteinase-9; NOTCH1, notch receptor 1; OTX2, orthodenticle homeobox 2; SHH, sonic hedgehog signaling molecule; STAR, steroidogenic acute regulatory protein; TNF, tumor necrosis factor; XIAP, X-linked inhibitor of apoptosis.

Network pharmacological analysis was conducted to elucidate the systems-level mechanism of cordycepin. We constructed and visualized the compound-target network of cordycepin using Cytoscape [48]. In the compound–target network, nodes represented cordycepin and its targets, whereas edges represented the predicted interactions between cordycepin and targets. The targets of cordycepin were colored to indicate the pathways related to breast cancer. The pathways related to breast cancer obtained from literature were as follows: apoptosis pathway, breast tumor kinase pathway, cyclin-dependent kinases pathway, estrogen signaling pathway, hedgehog signaling pathway, human epidermal growth factor receptor 2 (HER2) signaling pathway, mammalian target of rapamycin signaling pathway, notch signaling pathway, phosphoinositide 3-kinases (PI3K)- protein kinase B (Akt) signaling pathway, and Wingless and INT-1 (Wnt)/β-catenin signaling pathway [49,50]. The numbers of related targets for the apoptosis, estrogen signaling, notch signaling, and hedgehog signaling pathways were 5, 2, 2, and 2, respectively (Figure 2). B-cell lymphoma 2 (BCL2) was simultaneously related to the apoptosis, estrogen signaling, and hedgehog signaling pathways.

Next, we conducted GSEA to identify potential pathways that are significantly associated with the targets of cordycepin among the pathways related to breast cancer. The results showed that the apoptosis pathway had the highest overlap and lowest *p*-value for the targets of cordycepin. In addition, the hedgehog signaling, p53 signaling, and estrogen signaling pathways were significantly associated with the targets of cordycepin (Table 1).

### 3.3. Effects of the Cordyceps Militaris Concentrate and Cordycepin on Apoptosis

**Figure 3:**
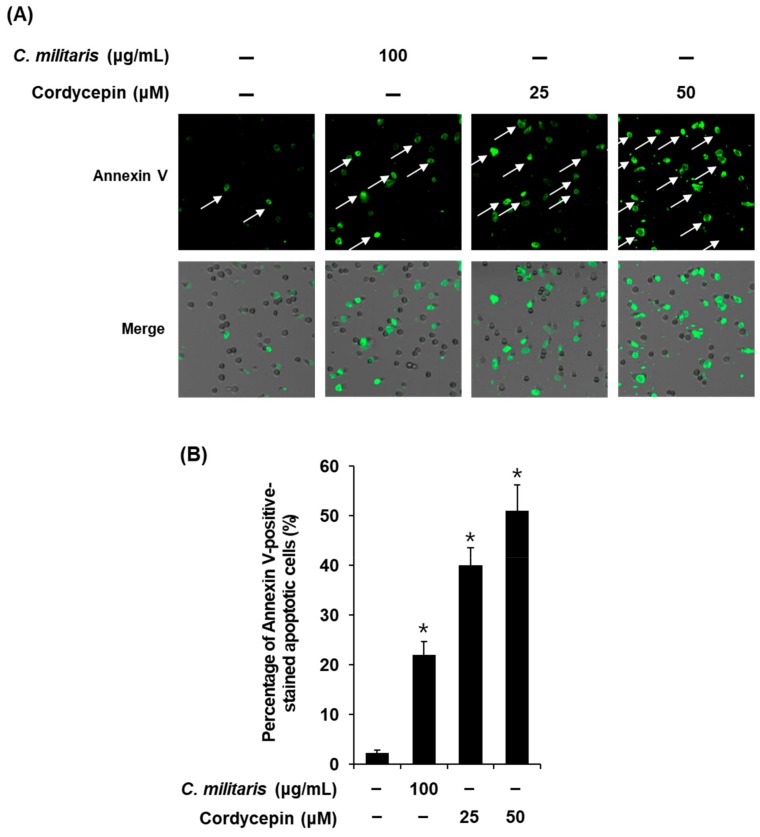
Effects of *Cordyceps militaris* concentrate (100 μg/mL) and cordycepin (25 and 50 μM) on apoptosis in MCF-7 breast cancer cells exposed for 24 h by image-based cytometric assay. (**A**) Representative images for apoptosis detection; (**B**) Percentage of Annexin V-positive-stained apoptotic cells. Data are the means of experiments performed in triplicate. Data are presented as the mean ± SD. and were analyzed using the Student’s *t*-test. *p < 0.05 versus non treated cells.

To verify the anticancer mechanisms identified through network pharmacological analysis, image-based cytometric assay and western blot analysis were performed for the *C. militaris* concentrate and cordycepin to evaluate their apoptotic effect on MCF-7 cells. To explore the effects of *C. militaris* and cordycepin on apoptotic cell death, MCF-7 cells were stained with Annexin V. After treatment with *C. militaris* concentrate (100 µg/mL) and cordycepin (25, 50 µM), apoptotic cells stained with enhanced Annexin V (green fluorescence) increased as compared to untreated normal cells (Figure 3A). The percentage of apoptotic cells was significantly increased to 22.50 ± 2.17%, 40.06 ± 3.49%, and 52.63 ± 2.56% after treatment with *C. militaris* concentrate (100 µg/mL) and cordycepin (25 µM, 50 µM), respectively (Figure 3B).

**Figure 4:**
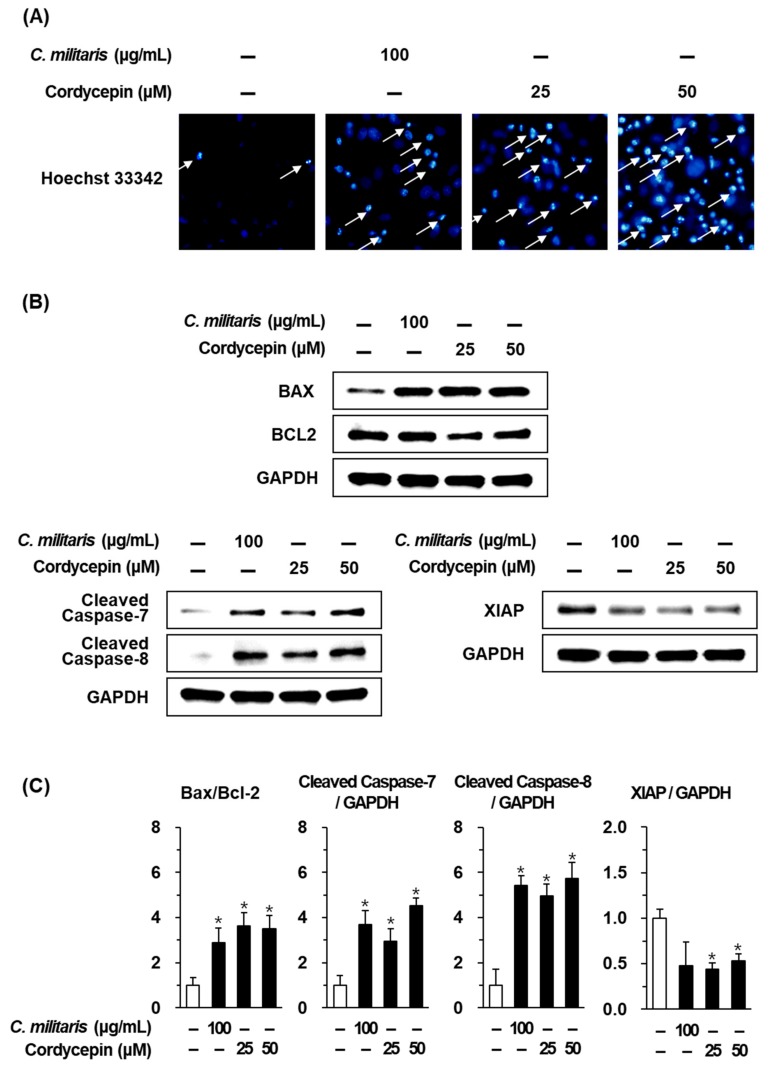
Effects of *Cordyceps militaris* concentrate (100 μg/mL) and cordycepin (25 and 50 μM) on apoptosis in MCF-7 breast cancer cells exposed for 24 h. (**A**) Results of Hoechst 33342 fluorescent staining of *Cordyceps militaris* and cordycepin to detect nuclear condensation of MCF-7 cells. (**B**) Protein expression of Bax, Bcl-2, cleaved caspase-7, cleaved caspase-8, XIAP and GAPDH. (**C**) Graph of relative protein expression. Data are the means of experiments performed in triplicate. Data are presented as the mean ± SD. and were analyzed using the Student’s *t*-test. *p < 0.05 versus non-treated cells.

In keeping with these results, DNA fragmentation identifying apoptotic cells was increased after treatment with *C. militaris* concentrate (100 µg/mL) and cordycepin (25, 50 µM). Blue fluorescence of Hoechst 33342 on these cells was brighter than that of untreated normal cells (Figure 4A). To identify the apoptotic mechanism of *C. militaris* concentrate and cordycepin, western blot analysis was performed. After treatment with *C. militaris* concentrate (100 µg/mL) and cordycepin (25, 50 µM), the Bax (pro-apoptotic Bcl-2 family)/Bcl-2 (anti-apoptotic Bcl-2 family) ratio was increased by more than two times. And cleavage of caspase-8, which is an apoptotic initiator, was significantly increased by more than five times. Furthermore, cleavage of caspase-7, an apoptotic effector caspase, was significantly increased by more than three times. Meanwhile, treatment of cordycepin inhibited XIAP, which is the X-linked inhibitor of apoptosis protein (Figure 4B).

## 4. Discussions

In our investigations on the *Cordyceps militaris* concentrate and cordycepin to examine in vitro anticancer activity on MCF-7 human breast cancer cells, we observed that both the *C. militaris* concentrate and cordycepin exhibited a significant cytotoxic effect on MCF-7 cells. The IC_50_ value of the latter was about five times lower than that of cisplatin, which is currently the most effective platinum-based drug for breast cancer [51]. The extract of *C. militaris* exhibited cytotoxic effects on the 4T1 murine breast cancer cell line in a recent study, and the extract (100, 200, and 400 µg/mL) inhibited cell viability in a concentration-dependent manner [52]. In addition, cordycepin (100 µM) has been reported to inhibit cell viability below 50% in MCF-7, MDA-MB 231, and MDA-MB-435 human breast cancer cells [53]. These results indicate that cordycepin exhibits cytotoxic effects on various types of breast cancer cell lines.

In the present study, the *C. militaris* concentrate and breast cancer cell line also suppressed cell proliferation of the MDA-MB-231 breast cancer cell line in a concentration-dependent manner (Figure S1). Although active concentrations on MDA-MB-231 were higher than on MCF-7, *C. militaris* concentrate and cordycepin also inhibited cell proliferation of the MDA-MB-231 breast cancer cell line. Meanwhile, 100 μg/mL of *C. militaris* concentrate did not affect the viabilities of LLC-PK1 pig kidney epithelial cells and human umbilical vein endothelial (HUVEC) cells, which are normal cell lines (Figure S2). Cordycepin also did not affect the viabilities of LLC-PK1 and HUVEC cells until 50 μM. We suggest that they are good candidates for targeting breast cancer.

To investigate the detailed cytotoxic effects, a phase contrast inverted microscope was used to evaluate the pattern of cell death in MCF-7 cells. Results of this observation indicated that apoptotic cell morphologies including membrane blebbing, cell shrinkage, and cell condensation were increased after treatment with *C. militaris* concentrate and cordycepin. These findings were consistent with the results of a previous study, which reported that cell debris and irregularly shaped cells were increased in MCF-7 cells after treatment with cordycepin (100 µM) [54]. However, these are hallmarks of apoptosis but not exact evidence [47]. Therefore, to evaluate apoptotic cell death under treatment with *C. militaris* concentrate and cordycepin in MCF-7 cells, Hoechst 33342 staining and image-based cytometric assays were used to distinguish apoptotic cells from non-apoptotic cells. Observation with a phase-contrast fluorescence microscope indicated that blue fluorescence from Hoechst 33342-staining of DNA as well as DNA fragmentation, which is a key characteristic of apoptosis [55], were also increased. In keeping with this result, apoptotic cells stained with enhanced Annexin V (green fluorescence) were increased after treatment with *C. militaris* concentrate and cordycepin.

To elucidate the systems-level mechanism, a compound–target network was constructed based on the predicted targets of cordycepin. The predicted targets were associated with the apoptosis, estrogen signaling, hedgehog signaling, and notch signaling pathways. In addition, GSEA was used to identify pathways that were significantly enriched in the target list of cordycepin. Among the pathways associated with breast cancer, the apoptosis pathway showed the greatest overlap, lowest *p*-value, and highest combined score.

To further investigate the changes in protein expression involving the apoptotic pathways, western blot analysis was used. The apoptotic pathways involved in the anticancer effects of cordycepin have been extensively studied in various cancers over a long period of time (about 60 years) [36]. Collectively, treatment with cordycepin increases the phosphorylation of terminal deoxynucleotidyl transferase (TdT) by protein kinase A (PKA) [56]. Treatment with cordycepin also induces apoptosis through the mitochondria-mediated intrinsic apoptotic pathways indicated by translocation of Bax from the cytosol to the mitochondria, release of cytochrome *c* from the mitochondria to the cytosol, and activation of caspase-9. Caspase-9 is activated by apaf-1 (apoptosome) and activates caspase-3 and -7 [57,58]. In addition, treatment with cordycepin induces death receptor-mediated extrinsic apoptotic pathways, as indicated by the activation of caspase-8, which stimulates two parallel cascades. One is the activation of caspase-3 and -7 and the other is the release of cytochrome *c* induced by the truncated Bid (pro-apoptotic Bcl-2 family), which translocates to the mitochondria and results in an increase in the Bax/Bcl-2 ratio [59,60]. Furthermore, treatment of cordycepin inhibited XIAP. XIAP is bound to caspase- 3, -7, and -9 and suppresses cell death by caspase overproduction [61].

**Figure 5:**
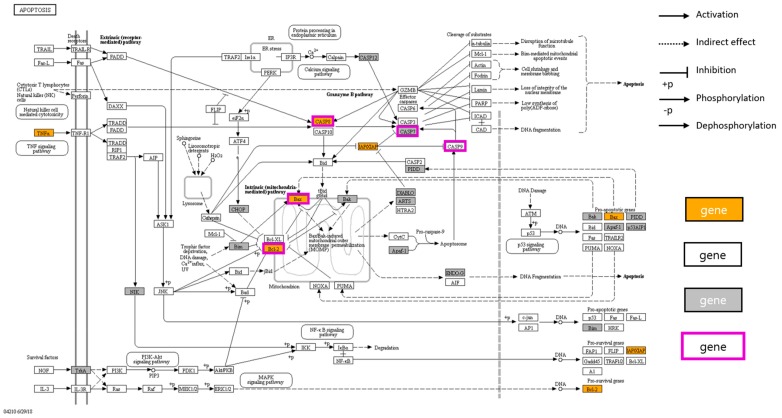
Apoptosis pathways (hsa04210) and targets of cordycepin. The pathway maps were constructed using KEGG mapper. The orange colored box represents a predicted target to interact cordycepin, the uncolored box represents a target that is expected to not interact, the gray colored box represents a target which is outside the predictable target list in TCM-mesh, and the purple-bordered box represents a validated target by western blot.

However, the MCF-7 cells used in our study were caspase-3 deficient [62,63]. Therefore, caspase-7 was confirmed as the apoptotic effector caspase. In the MCF-7 cells, *C. militaris* concentrate and cordycepin induced the cleavage of caspase-8, which are apoptotic initiator caspases. This resulted in the cleavage of caspase-7, an apoptotic effector caspase, and an increase in the Bax/Bcl-2 ratio. These results suggest that the *C. militaris* concentrate and cordycepin induced apoptosis via both mitochondrial-mediated intrinsic, and death receptor-mediated extrinsic apoptotic pathways. The targets of cordycepin related to the apoptosis pathway were summarized using KEGG Mapper (Figure 5).

Among the targets of cordycepin investigated by western blot analysis, three target genes were also found in the predicted target lists (*CASP8*, *Bax*, *Bcl-2* and *XIAP*). The accordance rate was significant (*p*-value < 0.001, hypergeometric test), which supports the reliability of the predicted results. Of the two mismatched targets, *CASP9* was predicted not to interact with cordycepin (incorrect prediction), and *CASP7* was not found in the predicted target list of TCM-mesh (unpredictable target).

In summary, we performed several in vitro experimental methods including cell viability assays, cell staining with Hoechst 33342, image-based cytometric assays, and western blot analysis to evaluate the apoptotic effects of the *C. militaris* concentrate and cordycepin on MCF-7 human breast cancer cells. The *C. militaris* concentrate and cordycepin exhibited potent cytotoxic effects on MCF-7 cells and could increase the cleavage of caspase-7 -8, and -9, and increase the Bax/Bcl-2 protein expression ratio in MCF-7 cells. Additionally, we applied network pharmacological analysis to predict potential targets and pathways of cordycepin and the targets related to the apoptosis pathway were validated in vitro.

## 5. Conclusions

In this study, the *C. militaris* concentrate cultured in brown rice and cordycepin isolated from *C. militaris* induced the cell death of MCF-7 human breast cancer cells. Network pharmacological analysis revealed a systems-level mechanism of cordycepin that could explain its suppressive effect on the proliferation of breast cancer cells, and the targets associated with the apoptosis pathway were verified by further experiments. This study provides a promising potential application not only for *C. militaris* cultured in brown rice itself but also for cordycepin isolated from *C. militaris* as a traditional medicine and alternative to chemotherapy in breast cancer. It also suggests that the strategy of integration of network pharmacology and experimental validation is a powerful tool to obtain a deep understanding of the mechanisms of natural products that act on multiple targets.

## Figures and Tables

**Table 1:** Significant enrichment pathway related to breast cancer by targets of cordycepin (adjusted *p*-value of ≤ 0.05).

Term	Overlap	Adjusted *p*-Value	Z-Score	Combined Score	Genes
Hedgehog signaling pathway	2/47	0.002	−55.763	410.509	*SHH, BCL2*
Apoptosis	5/143	>0.001	−4.979	81.932	*CASP8, BCL2, BAX, XIAP, TNF*
p53 signaling pathway	3/72	>0.001	−6.464	68.700	*CASP8, BCL2, BAX*
Estrogen signaling pathway	2/137	0.011	−0.946	4.964	*BCL2, MMP9*

## Supplementary Materials

The following are available online at https://www.mdpi.com/2218-273X/9/9/414/s1, Figure S1: Effects of *Cordyceps militaris* concentrate and cordycepin on MDA MB-231 breast cancer cell viability, Figure S2: Effects of *Cordyceps militaris* concentrate and cordycepin on viability of (A) LLC-PK1 pig kidney epithelial cells and (B) Human umbilical vein endothelial (HUVEC) cells.
