# Supplementary material for: The Inhibitory Effect of Cordycepin on the Proliferation of MCF-7 Breast Cancer Cells, and Its Mechanism: An Investigation Using Network Pharmacology-Based Analysis
Source: Biomolecules. 2019 Aug 26;9(9):414. doi: 10.3390/biom9090414 (PMC6770402; doi:10.3390/biom9090414)
Supplement: Supplementary file 1 [file biomolecules-09-00414-s001.pdf]

biomolecules

Supplementary Materials

Inhibitory effect of cordycepin on the proliferation of MCF-7 breast cancer cells and its mechanism investigation using network pharmacology-based analysis

Dahae Lee,[1,†] Won-Yung Lee,[2,†] Kiwon Jung,[3,†] Yong Sam Kwon,[4] Daeyoung Kim,[5] Gwi Seo Hwang,[2] Chang-Eop Kim,[2] Sullim Lee,[5,*] Ki Sung Kang[2,*]

[1] School of Pharmacy, Sungkyunkwan University, Suwon 16419, Republic of Korea; pjsldh@naver.com (D. L.)
[2] College of Korean Medicine, Gachon University, Seongnam 13120, Republic of Korea; wonyung21@naver.com (W.Y.L.); seoul@gachon.ac.kr (G.S.H.); eopchang@gachon.ac.kr (C.E.K.); kkang@gachon.ac.kr (K.S.K.)
[3] Institute of Pharmaceutical Sciences, College of Pharmacy, CHA University, Sungnam 13844, Korea; pharmj@cha.ac.kr (K.J.)
[4] Dong-A Pharmaceutical Co., LTD., Yongin 17073, Korea; vetkiss@donga.co.kr (Y.S.K.)
[5] Department of Life Science, College of Bio-Nano Technology, Gachon University, Seongnam, 13120, Republic of Korea; davekim@gachon.ac.kr (D.L.); sullimlee@gachon.ac.kr (S.L)
*Correspondence: sullimlee@gachon.ac.kr (S. L.) and kkang@gachon.ac.kr; Tel.: +82-42-750-5402 (K.S.K.)

† These authors contributed equally to this work.

Determination of Cell Viability

The cell viability of MDA-MB-231, LLC-PK1 and HUVEC cells in *C. militaris* concentrate and cordycepin was evaluated using an Ez-Cytox Cell Viability Assay Kit (Dail Lab Service Co., Seoul, Korea). Briefly, cells with a density of 1×10^4 cells/100 μL were seeded onto 96-well plates. After incubation for 24 h, *C. militaris* concentrate and cordycepin at various concentrations were added. After treatment for 24 h, 10 μL of Ez-Cytox solution was added and incubated for 30 min. The absorbance was measured at 450 nm (absorbance for live cells) in a microplate reader (PowerWave XS; Bio-Tek Instruments, Winooski, VT, USA).

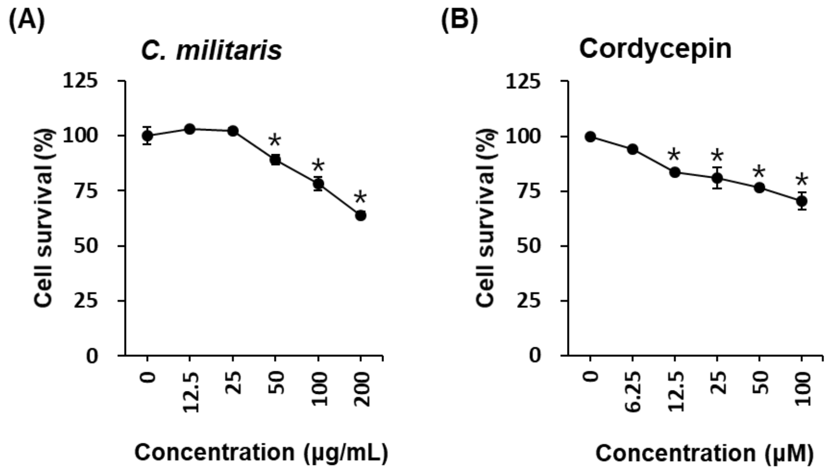

Figure S1. Effects of *Cordyceps militaris* concentrate and cordycepin on MDA-MB-231 breast cancer cell viability. Cytotoxic effects of (A) *C. militaris* concentrate and (B) cordycepin on MDA-MB-231 cells. Data are the means of experiments performed in triplicate. Data are presented as the mean ± S.D. and were analyzed using Student's t-test. *P < 0.05 versus non-treated cells.

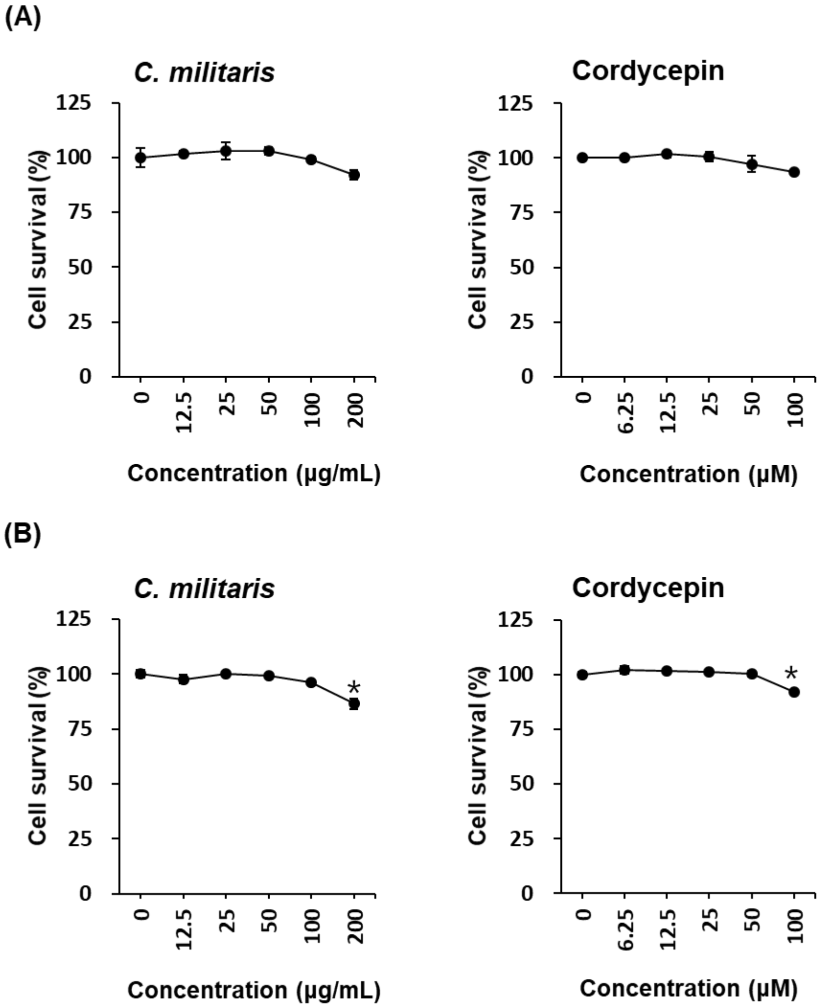

Figure S2. Effects of *Cordyceps militaris* concentrate and cordycepin on viability of (A) LLC-PK1 pig kidney epithelial cells and (B) Human umbilical vein endothelial (HUVEC) cells. Data are the means of experiments performed in triplicate. Data are presented as the mean ± S.D. and were analyzed using Student's t-test. *P < 0.05 versus non-treated cells.
